# Cloning, expression, and characterization of a peptidoglycan hydrolase from the *Burkholderia pseudomallei* phage ST79

**DOI:** 10.1186/s13568-016-0251-7

**Published:** 2016-09-15

**Authors:** Nittaya Khakhum, Umaporn Yordpratum, Atcha Boonmee, Unchalee Tattawasart, Jorge L. M. Rodrigues, Rasana W. Sermswan

**Affiliations:** 1 Department of Biochemistry and Melioidosis Research Center, Faculty of Medicine, Khon Kaen University, 123 Mitraparb Road, Khon Kaen, 40002 Thailand; 2 Department of Microbiology and Melioidosis Research Center, Faculty of Medicine, Khon Kaen University, 123 Mitraparb Road, Khon Kaen, 40002 Thailand; 3 Department of Microbiology, Faculty of Science, Khon Kaen University, 123 Mitraparb Road, Khon Kaen, 40002 Thailand; 4 Department of Land, Air and Water Resources, University of California, Davis, 3308 Plant and Environmental Sci. Bld., Davis, CA 95616 USA; 5 Environmental Genomics and Systems Biology Division, Lawrence Berkeley National Laboratory, Berkeley, CA 94720 USA

**Keywords:** Endolysins, Enzyme, Gene expression, Peptidoglycan

## Abstract

The lytic phage ST79 of *Burkholderia pseudomallei* can lyse a broad range of its host including antibiotic resistant isolates from within using a set of proteins, holin, lysB, lysC and endolysin, a peptidoglycan (PG) hydrolase enzyme. The phage ST79 endolysin gene identified as peptidase M15A was cloned, expressed and purified to evaluate its potential to lyse pathogenic bacteria. The molecular size of the purified enzyme is approximately 18 kDa and the in silico study cited here indicated the presence of a zinc-binding domain predicted to be a member of the subfamily A of a metallopeptidase. Its activity, however, was reduced by the presence of Zn^2+^. When *Escherichia coli* PG was used as a substrate and subjected to digestion for 5 min with 3 μg/ml of enzyme, the peptidase M15A showed 2 times higher in lysis efficiency when compared to the commercial lysozyme. The enzyme works in a broad alkaligenic pH range of 7.5–9.0 and temperatures from 25 to 42 °C. The enzyme was able to lyse 18 Gram-negative bacteria in which the outer membrane was permeabilized by chloroform treatment. Interestingly, it also lysed *Enterococcus* sp., but not other Gram-positive bacteria. In general, endolysin cannot lyse Gram-negative bacteria from outside, however, the cationic amphipathic C-terminal in some endolysins showed permeability to Gram-negative outer membranes. Genetically engineered ST79 peptidase M15A that showed a broad spectrum against Gram-negative bacterial PG or, in combination with an antibiotic the same way as combined drug methodology, could facilitate an effective treatment of severe or antibiotic resistant cases.

## Introduction

A bacteriophage or phage is the virus of bacteria that is very specific to its host. It can replicate, multiply after transfection and either lyse the host or become integrated into its genome (Golkar et al. 2014). Eastern Europe such as Poland and Georgia reported the phage as an alternative therapy for infectious diseases (Abedon et al. 2011). Phage therapy has also been evaluated in the United States (Ho 2001). The discovery of antibiotics changed the paradigm of curing infectious diseases and helped protect enormous numbers of people who suffered from bacterial infections in the twentieth century, however, the numbers of antibiotic-resistant bacteria have also increased (Golkar et al. 2014). Even though the post-antibiotic era is not at hand, the use of broad-spectrum antibiotics has proven to disturb the beneficial bacterial community in humans and animals, especially gut microbiota, that affect immunity (O’Hara and Shanahan 2006; Round and Mazmanian 2009). Therefore, the use of phages as an alternative treatment came under the spotlight again because the phage is highly specific, can overcome antibiotic resistant bacteria and are available in large quantities in nature.

The enzymes related to the lysis mechanism of the phages that are used to lyse the bacterial host during the release of their progeny have been studied and revealed the classical holin-endolysin lysis system found in phages of both Gram-positive and Gram-negative bacteria (Young et al. 2000). Holin is used to make pores in the cytoplasmic membrane that assists endolysin to access and cleave peptidoglycan to lyse the bacteria from the inside out. In addition, some phages of Gram-negative bacteria also have Rz/Rz1 or lysB/lysC accessory proteins that help in the lysis of the process (Berry et al. 2008). Endolysins have been considered as a new class of antibiotics as they can destroy the peptidoglycan (PG) of Gram-positive bacterial cell walls. Endolysin has five specific activities on PG which are muramidase (lysozyme), transglycosylase, glucosaminidase that digests *N*-acetylmuramic acids (NAM) and *N*-acetylglucosamine (NAG), amidase that digests NAM and peptides and endopeptidases digested within the peptide chain of PG (Borysowski et al. 2006). The enzymes or the phages themselves are extensively applied in several fields, for example, the food industry and biological control of unwanted bacteria (Ruyter et al. 1997) including pathogenic bacteria in medicine as it shows neither toxicity nor stimulates hyperimmune sera in the mouse model (Jado et al. 2003).

*Burkholderia pseudomallei* is a Gram-negative soil bacterium that causes severe septic infectious disease called melioidosis. The disease can be found in both humans and animals in endemic areas (Leelarasamee and Bovornkitti 1989). This pathogenic bacterium is intrinsically resistant to several antibiotics and it can produce high levels of biofilms that protect the bacterium from the killing by either antibiotics or the host immune response (Sawasdidoln et al. 2010; Pibalpakdee et al. 2012; Mongkolrob et al. 2015). The drug of choice is a third generation cephalosporin such as ceftazidime that needs long-term treatment to prevent relapse. Currently, there is no commercial vaccine available (Limmathurotsakul et al. 2015). Phages that have shown some specificity in lysing *B. pseudomallei* have been reported (Sariya et al. 2006; Yordpratum et al. 2011; Gatedee et al. 2011; Kvitko et al. 2012; Guang-Han et al. 2016). The genome of ST79, a novel lytic phage that lyses *B. pseudomallei* was sequenced and submitted to GenBank (GI:509141608) (manuscript in preparation). The lysis cassette of ST79 was also characterized (Khakhum et al. 2016) and its modified phages were shown to lyse a wide range of *B. pseudomallei* isolates and could significantly reduce biofilm formation of the bacteria especially at the early stage of attachment (Kulsuwan et al. 2015).

In this study, the peptidase M15A, known as endolysin or peptidoglycan hydrolase from the ST79 lytic phage that could lyse a broad spectrum of *B. pseudomallei* and other Gram-negative bacteria from within was cloned, expressed and characterized. More information on the enzymes and phages themselves could facilitate the application of them as adjunct standard antibiotic therapy for *B. pseudomallei*.

## Materials and methods

### Bacterial strains and ST79 phage

*Burkholderia pseudomallei* strain P37 was isolated from a blood sample from a patient admitted to Srinagarind Hospital, Faculty of Medicine, Khon Kaen University, Thailand. The *B. pseudomallei* lytic phage ST79, isolated from soil in the northeast of Thailand, was used as a source of the peptidase M15A for cloning (Yordpratum et al. 2011). *Escherichia coli* BL21 (DE3) was used as the host for cloning and protein expression processes (Thermo Fisher Scientific, Waltham, MA, USA). Eighteen Gram-negative bacteria, five of which were *E. coli* host strains; Top10, LMG194 (Invitrogen, CA, USA), DH5α, BL21 (DE3) and XL1-Blue (Thermo Fisher Scientific, Waltham, MA, USA), two *B. pseudomallei* isolates, P37 and G1; two *Burkholderia mallei* isolates, EY2233 and EY2237; *Burkholderia thailandensis* UE5 (kindly provided by MORU, Mahidol University, Thailand), *Klebsiella pneumoniae*, *Vibrio parahaemolyticus*, *Pseudomonas vasculitis*, *P. aeruginosa*, *Acinetobacter baumannii*, *Salmonella* gr. D, *Shigella* gr. D and *Citrobacter freundii* and seven Gram-positive bacteria included *Enterococcus* sp., *Streptococcus epidermidis*, *Staphylococcus aureus*, *Bacillus* sp., *Micrococcus* sp., β-*streptococcus* gr. B and *Corynebacterium diphtheria* were obtained from the Department of Microbiology, Faculty of Medicine, Khon Kaen University, Khon Kaen, Thailand and used in this study. All of bacterial strains and ST79 phage were deposited in culture collection belonging to World Data Centre For Microorganism (WDCM) as MRCKKU (registration number 1130). ST79 phage is available for research collaborators.

### Bioinformatic analysis

The peptidase M15A amino acid sequence (YP_008060500.1) from the ST79 phage genome (NC_02134.1) was submitted to BLASTP homology search (Altschul et al. 1997) in the NCBI database (http://www.ncbi.nlm.nih.gov/). The peptidase information resource and sequence analysis were performed by the MEROPS batch Blast tool (Rawlings and Morton 2008). The Interproscan 4 software v.4.8 (Zdobnov and Apweiler 2001) was used to analyze protein functional domains. The protein conserved domain and structure was predicted using Pfam (Finn et al. 2016) and SWISS-MODEL (Biasini et al. 2014).

### ST79 phage propagation and DNA extraction

The ST79 lytic phage was propagated in liquid culture using *B. pseudomallei* strain P37 as the propagating strain (Yordpratum et al. 2011). The phage DNA was extracted with a phenol–chloroform extraction method as described elsewhere (Sambrook and Russell 2001).

### Cloning and expression of peptidase M15A and Western blot analysis

The *peptidase M15A* gene was amplified from the ST79 genomic DNA by the polymerase chain reaction (PCR) using *in house* designed peptidase M15A forward primer (5′-TATAAAGAGCTCTATGCAGTTGACGGACCATTTC-3′) with *Sac*I restriction site (underlined sequence) and a peptidase M15A reverse primer (5′-ATAATAGGTACCTCATGCGCCCACCGTGTA-3′) with the *Kpn*I restriction site (underlined sequence). The PCR product was cloned into the pQE31 vector (Qiagen, Hilden, Germany) containing the N-terminal 6xhistidine tag and transformed into *E. coli* BL21 (DE3). *E. coli* cells containing the recombinant plasmid with *peptidase M15A* gene were propagated in Luria and Bertani (LB) medium containing 100 µg/ml ampicillin until mid-exponential phase (OD_600 nm_ = 0.6) and induced with 1 mM final concentration of isopropyl-β-d-thiogalactopyranoside (IPTG) (Sigma, St. Louis, Missouri, USA). Cells were further cultured for 4 h and collected as a pellet followed by sonication (10 cycles of 30 s pulses and 30 s rest at 200 Watts in an ice bath, MSE Soniprep 150, MSE, London, UK) in 5 ml lysis buffer (50 mM NaH_2_PO_4_, 300 mM NaCl, 10 mM imidazole pH 8.0). The protein supernatant and pellet fractions were separated by 15 % SDS-PAGE (Sambrook and Russell 2001) using the Novex Sharp Pre-Stained *Protein Standard* (Thermo Fisher Scientific, Waltham, MA, USA) as the protein molecular weight marker, then blotted onto nitrocellulose by a semi-dry blotting system (BioRad, CA, USA). The proteins on the membrane were detected by anti-6X His tag^®^ antibody (HRP) (Abcam, Cambridge, UK) and chemiluminescent SuperSignal West Pico Chemiluminescent Substrate (Thermo Fisher Scientific, Waltham, MA, USA).

### Protein purification

The recombinant *E. coli* BL21 (DE3) containing the *peptidase M15A* gene-plasmid was induced by IPTG and the His-tagged proteins inside *E. coli* cells were purified using Ni–NTA agarose (Qiagen, Hilden, Germany) by a gravity-flow chromatography method. SDS-PAGE was used to analyze the eluted fractions for the 18 kDa of the peptidase M15A protein. The fraction containing the protein was refolded by buffer exchanging with 25 mM sodium acetate buffer, pH 6.5 and filtered through the Amicon^®^ Ultra 10 K centrifugal filter device (Millipore, Darmstadt, Germany) according to the manufacturer’s instructions. The purified protein was quantified by the bicinchoninic acid assay (BCA assay) (Thermo Fisher Scientific, Waltham, MA, USA) according to the manufacturer’s instructions using a Spectronic 20D+ spectrophotometer (Thermo Fisher Scientific, Waltham, MA, USA).

### Zymogram analysis

The peptidase M15A enzyme activity was observed using zymogram or renaturing SDS-PAGE (Piuri and Hatfull 2006) with some modifications as follows: An overnight culture of *E. coli* XL1-Blue (0.2 % w/v) was autoclaved and added to the SDS-PAGE solution (15 % w/v) before polymerization. The purified peptidase M15A enzyme was mixed with 2× sample-refolding buffer (0.5 mM Tris–HCl pH 6.8, 20 % glycerol, 0.2 % bromophenol blue), then loaded into the SDS-PAGE containing *E. coli*. After electrophoresis, the gel was incubated at 37 °C for 16 h in 1 % triton X-100, 25 mM Tris–HCl pH 8.5 and washed once with water and stained for 3 h with 0.5 % methylene blue in 0.01 % KOH. The peptidoglycan hydrolase activity of peptidase M15A on *E. coli* lysate was observed as a clear zone.

### Enzyme activity

The outer membrane of *E. coli* XL1-Blue was permeabilized by chloroform to expose the peptidoglycan for digestion as described by Lavigne et al. (2004) with some modifications and used as the substrate for peptidase M15A. In brief, bacteria were grown until the mid-exponential phase (OD_600 nm_ = 0.6), then centrifuged at 4000*g* at 4 °C, for 15 min to collect the cell pellet. The outer membranes were permeabilized by chloroform-saturated 0.05 M Tris–HCl buffer, pH 7.5 and gently shaken at room temperature for 45 min. The permeabilized *E. coli* were then washed, resuspended in 10 mM phosphate buffer, pH 8.0 and adjusted to OD_600 nm_ = 0.6–1.0. The *E. coli* suspension of 270 µl was added into 96-well BD Falcon microplates (BD Bioscience, San Jose, CA, USA), then the purified peptidase M15A enzyme was added at the amount of 0.1 (0.3 μg/ml), 0.5 (1.6 μg/ml), 1 (3 μg/ml) and 5 μg (16 μg/ml) (30 µl) and the turbidity reduction at OD_600 nm_ was measured by a Gen5 microplate reader (Biotek, Vermont, USA) as the kinetic assay for 15 min (1 min time intervals). The chicken egg white lysozyme (Sigma, St. Louis, Missouri, USA) (3 μg/ml) was used as a positive control and 10 mM phosphate buffer, pH 8.0, was used as a negative control (Briers et al. 2007a).

### The effect of pH, temperature and divalent metal ions

To evaluate the effect of pH on enzymatic activity, permeabilized *E. coli* XL1-Blue cells were resuspended in 900 μl of 10 mM citrate buffer for pH 3.0–5.5, 10 mM phosphate buffer for pH 6.0–7.0 or 10 mM Tris–HCl buffer for pH 7.5–9.0 and then 100 μl of enzyme (5 μg) was added. The percentage of OD_600 nm_ reduction was determined after incubation at 30 °C for 15 min. The effect of temperatures at 25, 30, 37 and 42 °C on enzyme activity were tested in the same manner at pH = 8.0.

The effect of metal ion, particularly Zinc, on enzyme activity was observed using 100 μl (5 μg) of purified peptidase M15A mixed with 900 μl of permeabilized *E. coli* XL1-Blue cells (OD_600 nm_ ~ 1.0) in various concentrations of ZnCl_2_ (5, 10, 50 and 100 μM), MgCl_2_, MnCl_2_ or CaCl_2_ (100 and 1000 μM). The relative lytic activity was calculated as follows: {ΔOD_600 nm_ sample (endolysin added) − ΔOD_600 nm_ (buffer only)}/initial OD_600 nm_ (Plotka et al. 2015) and compared with the control without metal ions for 100 % of relative activity (Son et al. 2012).

### Spectrum of antibacterial lytic activity

A total of 18 Gram-negative and seven Gram-positive bacteria were tested for the peptidoglycan hydrolase spectrums. Gram-negative bacteria were permeabilized by chloroform and their peptidoglycans were used as substrates for enzyme digestion as previously described (Briers et al. 2007a). For Gram-positives, each strain was grown to mid-exponential phase then centrifuged at 3000*g* to collect cell pellets. The pellets were washed and resuspended in 10 mM phosphate buffer pH 8.0, the OD_600 nm_ to 0.6–1.0 was adjusted. To test the spectrum of antimicrobial lytic activity, 5 and 20 μg in 100 μl of purified peptidase M15A was added into 900 μl permeabilized cell suspensions of Gram-negative or cell suspension of Gram-positive. The score was estimated from % relative lytic activity after incubation at 30 °C for 15 min. The  % relative activity was defined as—(no lytic activity), + (1–30 %), ++ (31–60 %) and +++ (61–100 %) (Son et al. 2012).

### Statistical analysis

The data of OD_600 nm_ in the turbidity reduction test at different pHs and temperatures were analyzed by a Student’s *t* test and with a *p* value of  <0.05 considered as significant.

## Results

### Peptidase M15A sequence analysis

**Fig. 1:**
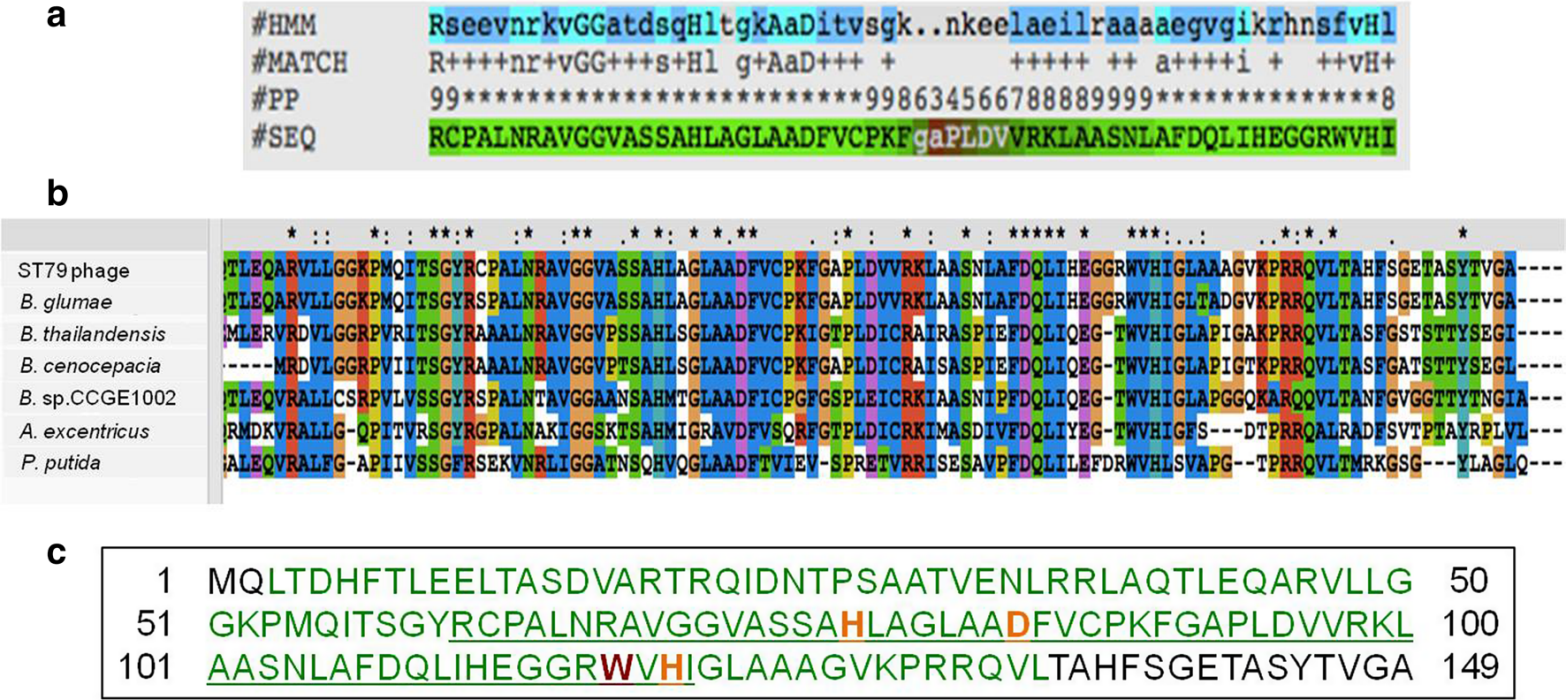
The bioinformatics analysis of ST79 peptidase M15A protein sequence. **a** The Peptidase_M15_3 conserved domain analyzed using Pfam; #HMM: consensus of the Hidden Markov Models (HMMs), Capital letters indicated the most conserved positions, #MATCH: the match between the query sequence and the HMM, ‘ + ’ indicates a positive score which can be interpreted as a conservative substitution, #PP: posterior probability, which is the degree of confidence in each individual aligned residue, 0 means 0–5 %, 1 means 5–15 % and so on and 9 means 85–95 %, ‘*’ means 95–100 % posterior probability, #SEQ: query sequence, ‘-’ indicated deletions in the query sequence with respect to the HMM, **b** Multiple alignment of peptidase protein sequences from the MEROPS database showed identity of peptidase M15A sequence from ST79 phage with peptidase enzymes from *B. glumae*, *B. thailandensis*, *B. cenocepacia*, *Burkholderia* spp. CCGE1002, *A. excentricus* and *P. putida* using the CLUSTAL X program. Conserved amino acids of all sequences were marked with an *asterisk*. **c** The full amino acids sequence analysis of ST79 peptidase M15A using MEROPS batch Blast. The peptidase unit was labeled in* green*, active site in* red*, metal ion ligand in* orange* and the conserved domain in* underlined letters*

The protein sequence of peptidase M15A from the lytic phage ST79 showed a conserved domain as Peptidase_M15_3 (PF08291) when analyzed by BLASTP and Pfam (Fig. 1a). The Interproscan 4 (version 4.8) protein functional domain indicated a Hedgehog signaling/DD-peptidase zinc-binding domain (SSF55166). The SWISS-MODEL protein structure prediction was matched with the 1lbu.1.A template, which was muramoyl-pentapeptide carboxypeptidase (MEROPS data). When the MEROPS batch Blast tool was used to detect peptidases and their non-peptidase homologues sequences in ST79 genome, it showed common amino acids among peptidases found in other bacterial genome, such as: *B. glumae*, *B. thailandensis*, *B. cenocepacia*, *Burkholderia* sp. CCGE1002, *Asticcacaulis excentricus* and *Pseudomonas putida* but not *B. pseudomallei* and *B. mallei* (Fig. 1b). This study’s analysis also identified a homologue catalytic domain from amino acid positions 3 to 134 (total 132 residues) with peptidase from *P. putida* (MER087996). The active site residues were located at the tryptophan (W117H) position and metal ligands at histidine (H77), aspartic acid (D84) and histidine (H119) (Fig. 1c). The analysis also indicated the presence of motifs HXXXXXXD and WXH, which were typical for peptidase M15 subfamily A.

### Expression, purification and zymogram analysis of the Peptidase M15A

**Fig. 2:**
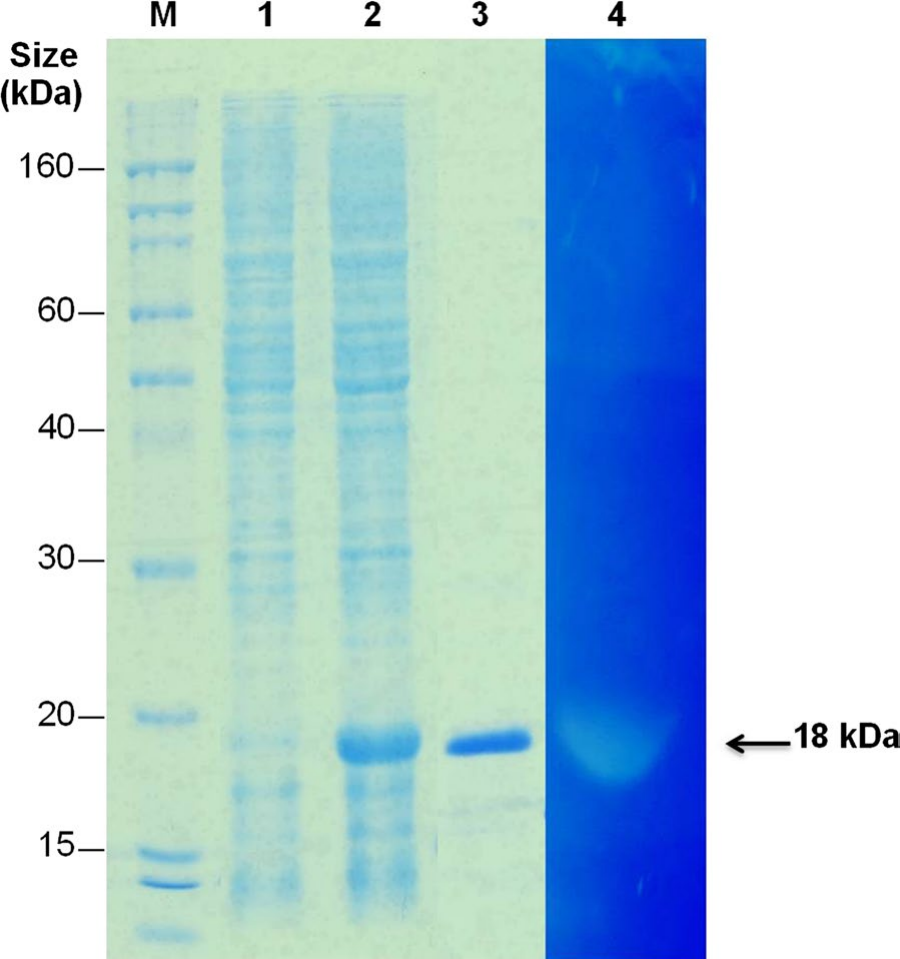
The SDS-PAGE analysis of proteins and zymogram analysis of the lytic activity of Peptidase M15A against *E. coli* peptidoglycan. The Coomassie Brilliant blue stained SDS-PAGE gel shows protein molecular weight marker (M), induced *E. coli* cells containing pQE31 vector (*lane 1*), induced cells containing peptidase M15A/pQE31 plasmid (*lane 2*), 5 μg of purified peptidase M15A protein (*lane 3*) and the zymogram that is stained with 0.1 % methylene blue in 0.01 % KOH of SDS-PAGE refolding gel (*lane 4*). The clear lysis of PG substrate by the enzyme on zymogram located at the same position of the over-expressed protein band in *lane 2* and purified enzyme in *lane 3*. The *arrow* indicates the 18 kDa lytic band of peptidase M15A

The *peptidase M15A* gene was successfully cloned and expressed in *E. coli* BL21 (DE3). The estimated molecular weight of purified peptidase M15A on SDS-PAGE was approximately 18 kDa (with 6xhis tag) (Fig. 2). For zymogram analysis, the peptidase M15A enzyme in the gel lysed the peptidoglycan substrate from *E. coli* in which it appeared as a transparent band (Fig. 2).

### Lytic activity test

**Fig. 3:**
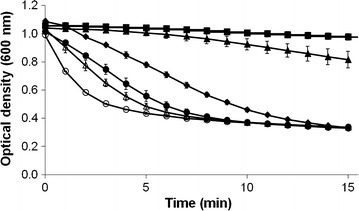
The turbidity reduction curve of peptidase activity against chloroform-treated *E. coli*. The peptidase M15A activity of various amounts was used to lyse chloroform *E. coli* XL1-Blue cell suspensions. The graph of turbidity reduction using peptidase M15A of 0.3 μg/ml (*filled triangle*), 1.6 μg/ml (*filled circle*), 3 μg/ml (*open triangle*) and 16 μg/ml (*open circle*) were evaluated. Phosphate buffer (*filled square*) was used as a negative control and 3 μg/ml of lysozyme (*filled diamond*) was used as positive control. Each point represents the mean of triplicate experiments and* error bars* indicate the standard deviation (SD)

At the concentration of 1.6 μg/ml onward at 5 min of digestion, the enzyme reduced the turbidity of permeabilized *E. coli* XL1-Blue more than the lysozyme. When longer times of 10 and 15 min were observed, the turbidities from each concentration including lysozyme were similar. When the same concentrations of lysozyme and purified peptidase M15A (3 μg/ml) were compared at 5 min of digestion, the lysozyme gave a 22 % relative lytic activity while the peptidase M15A resulted in 52 %. The purified peptidase M15A digested the substrate approximate 2 times more than the lysozyme at this point (Fig. 3).

### Effect of pH, temperature and divalent metal ions on the enzyme activity

**Fig. 4:**
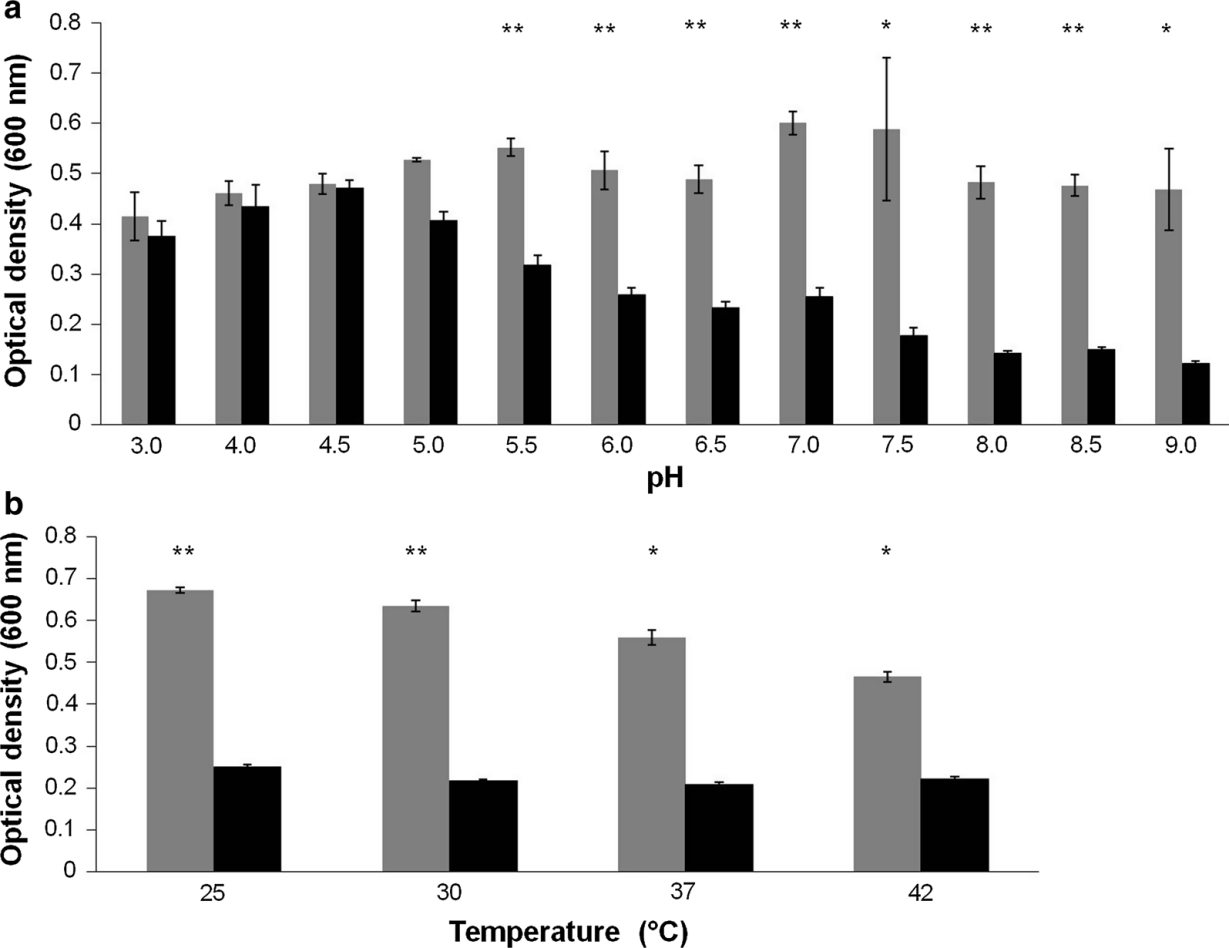
The effect of pH and temperature on the peptidase M15A lytic activity against *E. coli* XL1-Blue peptidoglycan. The optical density at 600 nm of cell suspensions was used to observe the effects of pH **a** and temperature **b** on the peptidase M15A lytic activity against *E. coli* XL1-Blue peptidoglycan. The* grey bars* show negative controls without enzyme and* black bars* indicate the reaction with enzyme. *p* value <0.05 (*) and 0.01 (**) indicate the significance. Each* column *represents the mean of triplicate experiments and* error bars* indicate SD

**Fig. 5:**
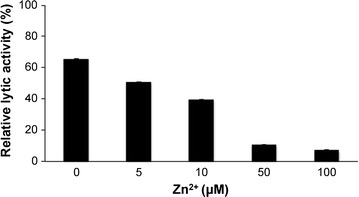
The percentage of *E. coli* XL1-Blue peptidoglycan reduction when treated with peptidase M15A in the presence of Zn^2+^. The effect of Zn^2+^ on peptidase M15A activity was calculated from the difference between optical densities at 600 nm of enzyme treated cells and untreated cells. Each* column* represents the mean of triplicate experiments and* error bars* indicate SD

The peptidase M15A showed highest the activity at pH 7.5–9.0 with relative activity above 60 % (Fig. 4a). The enzyme could work in broad temperature ranges of 25, 30, 37 and 42 °C (Fig. 4b). The enzyme activity was decreased when Zn^2+^ concentrations were increased (Fig. 5). On the other hand, the 100 μM of Mg^2+^ and Mn^2+^ could only increase approximately 10 % of relative activity. When the 1000 μM concentration of Mg^2+^ and Mn^2+^ was used, the enzyme activity was reduced to 50.3 and 65.6 %. The Ca^2+^ ion showed little effect on the enzyme activity as seen by an approximately 6 % increase with 100 μM of the ion and a 5 % decrease when 1000 μM was used (Table 1).

**Table 1:** The effect of divalent metal ions on lytic activity of peptidase M15A enzyme

Relative lytic activity (%)^a^		
Without metal ions (control)	100.0 ± 1.98	
Metal ions	100 μM	1000 μM
Mg^2+^	110.0 ± 2.6	50.3 ± 6.8
Ca^2+^	106.1 ± 5.4	95.3 ± 4.2
Mn^2+^	111.9 ± 0.4	65.6 ± 21.5

^a^The activities are shown as percentages in relation to the non-treated peptidase M15A control. Values represent the mean ± standard deviation of triplicate experiments

### Spectrum of antibacterial lytic activity

Eighteen chloroform permeabilized Gram-negative and seven Gram-positive bacteria were used for susceptibility tests against 5 and 20 μg of the peptidase M15A (Table 2). The enzyme lysed the peptidoglycan from all Gram-negative bacteria investigated. The enzyme effectively lysed *E. coli*, *K. pneumoniae*, *Shigella* gr. D and *C. freundii* and moderately lysed *Burkholderia* spp.*, V. parahaemolyticus, P. vasculitis, P. aeruginosa, A. baumannii* and *Salmonella* gr. D (36–82 %). For Gram-positive, it only lyzed *Enterococcus* sp. (49 %).

**Table 2:** Spectrum of antibacterial lytic activity against Gram-negative and Gram-positive bacteria

Bacterial strains	% Relative lytic activity^a^
Gram–negative bacteria	
*E. coli* Top10	+++
*E.* DH5α	+++
*E. coli* BL21 (DE3)	+++
*E. coli* LMG194	+++
*E. coli* XL1-Blue	+++
*B. pseudomallei* P37	++
*B. pseudomallei* G1	++
*B. mallei* EY2233	++
*B. mallei* EY2237	++
*B. thailandensis* UE5	++
*K. pneumoniae*	+++
*V. parahaemolyticus*	++
*P. vasculitis*	++
*P. aeruginosa*	++
*A. baumannii*	++
*Salmonella* gr. D	++
*Shigella* gr. D	+++
*C. freundii*	+++
Gram–positive bacteria	
*Enterococcus* sp.	++
*S. epidermidis*	–
*S. aureus*	–
*Bacillus* sp.	–
*Micrococcus* sp.	–
*β*-*streptococcus* gr. B	–
*C. diphtheriae*	–

^a^% Relative lytic activity was calculated from different optical densities at 600 _nm_ between the samples treated with buffer and enzyme. The score is indicated as: –no activity and +, ++, +++ indicating the different ranges including 1–30 %, 31–60 % and 61–100 %

## Discussion

The increase in antibiotic-resistant bacteria makes the use of possible phage therapy as an alternative treatment for bacterial infections as one of multiple options for treatment. Similar to the concept of a mixed viral vaccine, phages also could be used as a portion of a cocktail for broad host range lysis and more phage could be added into suit the resistance situation (Chan et al. 2013). PG is the major component of Gram-positive bacteria and also the lining under the outer membrane of Gram-negative bacteria. Endolysins are a group of PG hydrolyzing enzymes well characterized in phages for their function on the release of the progeny out of the bacterial host during the lytic cycle. The information on the specificity of the phage and its enzymes against bacteria could facilitate their use safely.

A novel lytic phage ST79 and its modified phage that lyses *B. pseudomallei* has been reported to effectively lyse a broad range of the bacterium (Yordpratum et al. 2011; Kulsuwan et al. 2015). ST79 endolysin-like protein, peptidase M15A, was identified from the phage genome sequence and predicted to contain 149 amino acids (approximately 16 kDa) with catalytic but not the binding domain (Khakhum et al. 2016). This was similar to other lysins from phages that infect Gram-negative bacterial hosts, which contain single catalytic domains with a molecular mass of 15–20 kDa (Nelson et al. 2012). Endolysins KZ144 and EL188 from a *Pseudomonas* phage, however, were shown to contain both lytic and N-terminal binding domains (Briers et al. 2007b).

The peptidase M15A amino acid sequence analyzed by MEROPS showed a high identity with conserved amino acid sequences of the peptidase M15 subfamily A that is typical for metallopeptidases with a metal binding part (Rawlings and Morton 2008). A similar prediction was observed with BLASTP and Pfam results, detecting a conserved domain of peptidase M15_3_superfamily. The peptidase M15A from ST79 phage has 132 amino acid residues with active sites containing a Zn^2+^ ion-binding site at the following amino acids: His77, Asp84 and His199. The peptidase enzyme of a *Streptomyces* phage also contains His154, Asp161 and His197 of the D-Ala-D-Ala carboxypeptidase zinc specific cleavage site. When the Zn^2+^ ion was present, the activity of peptidase M15A from ST79 phage was inhibited while that of *Streptomyces* works more effectively (Courvalin 2006). Likewise, the Zn^2+^ inhibition effect is also found in the phage T5 endolysin, in which a 10 mM concentration completely inactivated the enzyme immediately after addition. It was also observed that this specific endolysin requires Ca^2+^ instead of Zn^2+^ or Mn^2+^ at the stage of the phage developmental cycle (Mikoulinskaia et al. 2009). Activity of the peptidase M15A was inhibited with addition of 100 μM Zn^2+^ (10 % of relative activity remain) while 100 μM of Mg^2+^, Ca^2+^ and Mn^2+^ caused a slightly increased enzyme activity. When 1000 μM of Mg^2+^, Mn^2+^ and Ca^2+^ were used, the activity was decreased. On the contrary, Zn^2+^ and Mn^2+^ are required for full enzymatic activity of LysB4 endolysin from the *B. cereus*-infecting phage B4 (Son et al. 2012) and this requirement is also seen in Ply500 endolysin from the *Listeria monocytogenes* phages (Loessner et al. 1995).

Characterization of some biochemical properties of the peptidase M15A showed a broad range of optimal pHs varying from 7.5 to 9.0 (% relative lytic activity >60 %), which is in alkalophilic range similar to other previously reported peptidases as observed for phage T5 endolysin (optimum pH 8.5) (Mikoulinskaia et al. 2009), lysB4 (pH 8.0–10.0) (Son et al. 2012), transglycosylase endolysin of the phage SPN1S (pH 7.0–10.5), and the highly thermostable Ts2631 amidase endolysin from the *Thermus scotoductus* phage vB_tsc2631 (7.0–11.0) (Plotka et al. 2015). The Peptidase M15A worked at 25–37 °C and also 42 °C which is the optimum temperature for cultivation of *B. pseudomallei* in the laboratory (Chen et al. 2003; Palasatien et al. 2008). The enzyme from phages mostly works in an alkalophilic pH.

In this study, all the chloroform permeabilized Gram-negative bacteria were prepared and used as a substrate to test the specificity of endolysin against PG (Briers et al. 2007a). The PG from 18 strains of Gram-negative bacteria including drug resistant strains *B. pseudomallei* G1, *B. mallei* EY2233 can be lysed by ST79 peptidase M15A. The enzyme may be more specific to peptidoglycans of Gram-negatives as the enzyme can lyse only *Enterococcus* sp. among eight Gram-positive bacteria tested. The amino acid composition and sequence of PG in Gram-negative bacteria is known to have a low variation and the PG type belong to A1γ. Even though PG from *C. diphtheriae* contains A1γ as in Gram-negative bacteria (Schleifer and Kandler 1972), its cell wall is distinct from others with a predominance of meso-diaminopimelic acid in the murein wall and multiple repetitions of arabinogalactan (Besserer et al. 2006). The PG is A1γ type, similar to *Bacillus* sp., but has modification like deacetylation and resists lysozyme digestion (Davis and Weiser 2011). Both of them were resistant to the ST79 peptidase M15A. For other Gram-positive bacteria, *S. epidermidis*, *S. aureus, Micrococcus* spp. and *β*- *streptococcus* group B, their PG belongs to the A3α type (Schleifer and Kandler 1972), preventing lysis by the peptidase M15A. Interestingly, *Enterococcus* sp., which is a Healthcare–Associated Infections (HAI) bacterium, was effectively lysed by ST79 peptidase M15A. The enzyme could act on the D-Ala-D-Ala termini of *Enterococcus* cell wall peptidoglycan (Arthur et al. 1996). Therefore, the action of the ST79 peptidase M15A may be specific to the peptidoglycan type A1γ of Gram-negative bacteria and to the Gram-positive *Enterococcus* sp.

Even though, in general, endolysin cannot attack the PG which is located under the outer membrane in Gram-negative bacteria, but permeabilized the outer membrane for example with 10 mM EDTA in combination with 50 mg/ml of the *Pseudomonas* endolysin EL188 can decrease the viable *P. aeruginosa* cells by 3 or 4 orders of magnitude in 30 min (Briers et al. 2011). Interestingly, LysAB2, the endolysin from *A. baumannii* phage ϕAB2 was reported to have an antibacterial effect against both Gram-negative (*A. baumannii*, *E. coli*, *Salmonella enterica*) and Gram-positive (*Streptococcus sanguis*, *S. aureus, B. subtilis*) strains (Lai et al. 2011). LysAB2 contains a C-terminal amphipathic region that is necessary for the antibacterial activity as also reported in the Lys1521 endolysin from a *B. amyloliquefaciens* phage that contains two cationic C-terminal regions. The cationic region was demonstrated to permeabilize the outer membrane of *P. aeruginosa* (Muyombwe et al. 1999). It is therefore possible to genetically engineer endolysin to have the cationic C-terminal regions to lyse Gram-negative bacteria from outside the same way as suggested by Nelson et al. (2012).

Lysozyme, also known as muramidase, is a well-known endolysin enzyme that is generally used as a standard for comparisons of endolysin activity. For example, *A. baumannii* phage phiAB2 endolysin activity was only 30 % activity of chicken egg white lysozyme (Lai et al. 2011). When the ST79 peptidase M15A activity was compared with chicken egg white lysozyme, it could reduce PG substrate approximately 2 times higher in lysis efficiency than lysozyme. Nevertheless, the transglycosylase SPN1S endolysin from *Salmonella typhimurium*-infecting phage can reduce 50 % OD of a 1 ml EDTA pretreated cell suspension when only 50 nanograms was used. This phage enzyme, therefore, has approximately 30 times higher activity than lysozyme (Lim et al. 2012) and also more than ST79 peptidase M15A.

In conclusion, ST79 peptidase M15A is specific to A1γ PG and cleaves PG at the peptide chains. The enzyme can work in a broad alkaligenic range and temperature, has higher activity when compared to lysozyme and is also active against a broad range of Gram-negative bacterial PG and also *Enterococcus* sp. that make the enzyme an outstanding one for further development. The cationic amphipathic C-terminal in some endolysins that showed permeability to Gram-negative bacteria may be genetically engineered into ST79 peptidase M15A and used as an adjunct to standard antibiotic therapy for *B. pseudomallei* infection. The combination of a compound that permeabilizes *B. pseudomallei*’s outer membrane with the enzyme that attacks PG may provide a more effective treatment in severe or drug resistant cases. Intensive investigation is, however, definitely required.

## Abbreviations

PG: peptidoglycan
NAM: *N*-acetylmuramic acids
NAG: *N*-acetylglucosamine
NCBI: National Center for Biotechnology Information
PCR: polymerase chain reaction
LB: Luria and Bertani medium
IPTG: isopropyl-β-d-thiogalactopyranoside
SDS-PAGE: sodium dodecyl sulfate polyacrylamide gel electrophoresis
HAI: Healthcare–Associated Infections
EDTA: Ethylenediaminetetraacetic acid
OD: optical density

## References

[CR1] Abedon ST, Kuhl SJ, Blasdel BG, Kutter EM (2011). Phage treatment of human infections. Bacteriophage.

[CR2] Altschul SF, Madden TL, Schäffer AA, Zhang J, Zhang Z, Miller W, Lipman DJ (1997). Gapped BLAST and PSI-BLAST: a new generation of protein database search programs. Nucleic Acids Res.

[CR3] Arthur M, Reynolds P, Courvalin P (1996). Glycopeptide resistance in enterococci. Trends Microbiol.

[CR4] Berry J, Summer EJ, Struck DK, Young R (2008). The final step in the phage infection cycle: the Rz and Rz1 lysis proteins link the inner and outer membranes. Mol Microbiol.

[CR5] Besserer A, Puech-Pagès V, Kiefer P, Gomez-Roldan V, Jauneau A, Roy S, Portais J-C, Roux C, Bécard G, Séjalon-Delmas N (2006). Strigolactones stimulate arbuscular mycorrhizal fungi by activating mitochondria. PLoS Biol.

[CR6] Biasini M, Bienert S, Waterhouse A, Arnold K, Studer G, Schmidt T, Kiefer F, Cassarino TG, Bertoni M, Bordoli L, Schwede T (2014). SWISS-MODEL: modelling protein tertiary and quaternary structure using evolutionary information. Nucleic Acids Res.

[CR7] Borysowski J, Weber-Dabrowska B, Górski A (2006). Bacteriophage endolysins as a novel class of antibacterial agents. Exp Biol Med (Maywood).

[CR8] Briers Y, Lavigne R, Volckaert G, Hertveldt K (2007). A standardized approach for accurate quantification of murein hydrolase activity in high-throughput assays. J Biochem Biophys Meth.

[CR9] Briers Y, Volckaert G, Cornelissen A, Lagaert S, Michiels CW, Hertveldt K, Lavigne R (2007). Muralytic activity and modular structure of the endolysins of *Pseudomonas aeruginosa* bacteriophages phiKZ and EL. Mol Microbiol.

[CR10] Briers Y, Walmagh M, Lavigne R (2011). Use of bacteriophage endolysin EL188 and outer membrane permeabilizers against *Pseudomonas aeruginosa*. J Appl Microbiol.

[CR11] Chan BK, Abedon ST, Loc-Carrillo C (2013). Phage cocktails and the future of phage therapy. Future Microbiol.

[CR12] Chen YS, Chen SC, Kao CM, Chen YL (2003). Effects of soil pH, temperature and water content on the growth of *Burkholderia pseudomallei*. Folia Microbiol.

[CR13] Courvalin P (2006). Vancomycin resistance in gram-positive cocci. Clinic Infect Dis.

[CR14] Davis KM, Weiser JN (2011). Modifications to the peptidoglycan backbone help bacteria to establish infection. Infect Immun.

[CR15] Finn RD, Coggill P, Eberhardt RY, Eddy SR, Mistry J, Mitchell AL, Potter SC, Punta M, Qureshi M, Sangrador-Vegas A, Salazar GA, Tate J, Bateman A (2016). The Pfam protein families database: towards a more sustainable future. Nucleic Acids Res.

[CR41] de Ruyter PGGA, Kuipers OP, Meijer WC, de Vos WM (1997). Food-grade controlled lysis of *Lactococcus lactis* for accelerated cheese ripening. Nat Biotech.

[CR16] Gatedee J, Kritsiriwuthinan K, Galyov EE, Shan J, Dubinina E, Intarak N, Clokie MRJ, Korbsrisate S (2011). Isolation and characterization of a novel podovirus which infects *Burkholderia pseudomallei*. Virol J.

[CR17] Golkar Z, Bagasra O, Pace DG (2014). Bacteriophage therapy: a potential solution for the antibiotic resistance crisis. J Infect Dev Ctries.

[CR18] Guang-Han O, Leang-Chung C, Vellasamy KM, Mariappan V, Li-Yen C, Vadivelu J (2016). Experimental Phage Therapy for *Burkholderia pseudomallei* Infection. PLoS One.

[CR19] Ho K (2001). Bacteriophage therapy for bacterial infections: rekindling a memory from the pre-antibiotics era. Perspect Biol Med.

[CR20] Jado I, López R, García E, Fenoll A, Casal J, García P, Network on behalf of the SPIS (2003). Phagelytic enzymes as therapy for antibiotic-resistant *Streptococcus pneumoniae* infection in a murine sepsis model. J Antimicrob Chemother.

[CR21] Khakhum N, Yordpratum U, Boonmee A, Tattawasart U, Rodrigues JL, Sermswan RW (2016). Identification of the *Burkholderia pseudomallei* bacteriophage ST79 lysis gene cassette. J Appl Microbiol.

[CR22] Kulsuwan R, Wongratanacheewin S, Wongratanacheewin RS, Yordpratum U, Tattawasart U (2015). Lytic capability of bacteriophages (Family Myoviridae) on *Burkholderia pseudomallei*. Southeast Asian J Trop Med Public Health.

[CR23] Kvitko BH, Cox CR, DeShazer D, Johnson SL, Voorhees KJ, Schweizer HP (2012). φX216, a P2-like bacteriophage with broad *Burkholderia pseudomallei* and *B*. *mallei* strain infectivity. BMC Microbiol.

[CR24] Lai M-J, Lin N-T, Hu A, Soo P-C, Chen L-K, Chen L-H, Chang K-C (2011). Antibacterial activity of *Acinetobacter baumannii* phage ϕAB2 endolysin (LysAB2) against both gram-positive and gram-negative bacteria. App Microbiol biotechnol.

[CR25] Leelarasamee A, Bovornkitti S (1989). Melioidosis: review and update. Rev Infect Dis.

[CR26] Lavigne R, Briers Y, Hertveldt K, Robben J, Volckaert G (2004). Identification and characterization of a highly thermostable bacteriophage lysozyme. Cell Mol Life Sci.

[CR27] Lim J-A, Shin H, Kang D-H, Ryu S (2012). Characterization of endolysin from a *Salmonella Typhimurium*-infecting bacteriophage SPN1S. Res Microbiol.

[CR28] Limmathurotsakul D, Funnell SGP, Torres AG, Morici LA, Brett PJ, Dunachie S, Atkins T, Altmann DM, Bancroft G, Peacock SJ (2015). Consensus on the development of vaccines against naturally acquired melioidosis. Emerg Infect Dis.

[CR29] Loessner MJ, Wendlinger G, Scherer S (1995). Heterogeneous endolysins in *Listeria monocytogenes* bacteriophages: a new class of enzymes and evidence for conserved holin genes within the siphoviral lysis cassettes. Mol Microbiol.

[CR30] Muyombwe A, Tanji Y, Unno H (1999). Cloning and expression of a gene encoding the lytic functions of *Bacillus amyloliquefaciens* phage: evidence of an auxiliary lysis system. J Biosci Bioeng.

[CR31] Mikoulinskaia GV, Odinokova IV, Zimin AA, Lysanskaya VY, Feofanov SA, Stepnaya OA (2009). Identification and characterization of the metal ion-dependent l-alanoyl-d-glutamate peptidase encoded by bacteriophage T5. FEBS J.

[CR32] Mongkolrob R, Taweechaisupapong S, Tungpradabkul S (2015). Correlation between biofilm production, antibiotic susceptibility and exopolysaccharide composition in *Burkholderia pseudomallei* bpsI, ppk, and rpoS mutant strains. Microbiol Immunol.

[CR33] Nelson DC, Schmelcher M, Rodriguez-Rubio L, Klumpp J, Pritchard DG, Dong S, Donovan DM (2012). Endolysins as antimicrobials. Adv Virus Res.

[CR34] O’Hara AM, Shanahan F (2006). The gut flora as a forgotten organ. EMBO Rep.

[CR35] Palasatien S, Lertsirivorakul R, Royros P, Wongratanacheewin S, Sermswan RW (2008). Soil physicochemical properties related to the presence of *Burkholderia pseudomallei*. Trans R Soc Trop Med Hyg.

[CR36] Pibalpakdee P, Wongratanacheewin S, Taweechaisupapong S, Niumsup PR (2012). Diffusion and activity of antibiotics against *Burkholderia pseudomallei* biofilms. Int J Antimicrob Agents.

[CR37] Piuri M, Hatfull GF (2006). A peptidoglycan hydrolase motif within the mycobacteriophage TM4 tape measure protein promotes efficient infection of stationary phase cells. Mol Microbiol.

[CR38] Plotka M, Kaczorowska AK, Morzywolek A, Makowska J, Kozlowski LP (2015). Biochemical characterization and validation of a catalytic site of a highly thermostable Ts2631 endolysin from the *Thermus scotoductus* phage vB_Tsc2631. PLoS One.

[CR39] Rawlings ND, Morton FR (2008). The MEROPS batch BLAST: a tool to detect peptidases and their non-peptidase homologues in a genome. Biochimie.

[CR40] Round JL, Mazmanian SK (2009). The gut microbiota shapes intestinal immune responses during health and disease. Nat Rev Immunol.

[CR42] Sambrook J, Russell DW (2001). Molecular Cloning: a laboratory manual.

[CR43] Sariya L, Prempracha N, Keelapan P, Chittasophon N (2006). Bacteriophage isolated from *Burkholderia pseudomallei* causes phenotypic changes in *Burkholderia thailandensis*. Sci Asia.

[CR44] Sawasdidoln C, Taweechaisupapong S, Sermswan RW, Tattawasart U, Tungpradabkul S, Wongratanacheewin S (2010). Growing *Burkholderia pseudomallei* in biofilm stimulating conditions significantly induces antimicrobial resistance. PLoS One.

[CR45] Schleifer KH, Kandler O (1972). Peptidoglycan types of bacterial cell walls and their taxonomic implications. Bacteriol Rev.

[CR46] Son B, Yun J, Lim J-A, Shin H, Heu S, Ryu S (2012). Characterization of LysB4, an endolysin from the *Bacillus cereus*-infecting bacteriophage B4. BMC Microbiol.

[CR47] Yordpratum U, Tattawasart U, Wongratanacheewin S, Sermswan RW (2011). Novel lytic bacteriophages from soil that lyse *Burkholderia pseudomallei*. FEMS Microbiol Lett.

[CR48] Young I, Wang I, Roof WD (2000). Phages will out: strategies of host cell lysis. Trends Microbiol.

[CR49] Zdobnov EM, Apweiler R (2001). InterProScan–an integration platform for the signature-recognition methods in InterPro. Bioinformatics.

